# A Computed Microtomography Method for Understanding Epiphyseal Growth Plate Fusion

**DOI:** 10.3389/fmats.2017.00048

**Published:** 2018-01-23

**Authors:** Katherine A. Staines, Kamel Madi, Behzad Javaheri, Peter D. Lee, Andrew A. Pitsillides

**Affiliations:** 1 School of Applied Sciences, Edinburgh Napier University, Edinburgh, United Kingdom; 2 School of Materials, The University of Manchester, Manchester, United Kingdom; 3 Comparative Biomedical Sciences, Royal Veterinary College, London, United Kingdom

**Keywords:** growth plate, mouse, bone, synchrotron, computed tomography, fusion

## Abstract

The epiphyseal growth plate is a developmental region responsible for linear bone growth, in which chondrocytes undertake a tightly regulated series of biological processes. Concomitant with the cessation of growth and sexual maturation, the human growth plate undergoes progressive narrowing, and ultimately disappears. Despite the crucial role of this growth plate fusion “bridging” event, the precise mechanisms by which it is governed are complex and yet to be established. Progress is hindered by the current methods for growth plate visualization; these are invasive and largely rely on histological procedures. Here, we describe our non-invasive method utilizing synchrotron X-ray computed microtomography for the examination of growth plate bridging, which ultimately leads to its closure coincident with termination of further longitudinal bone growth. We then apply this method to a dataset obtained from a benchtop micro computed tomography scanner to highlight its potential for wide usage. Furthermore, we conduct finite element modeling at the micron-scale to reveal the effects of growth plate bridging on local tissue mechanics. Employment of these 3D analyses of growth plate bone bridging is likely to advance our understanding of the physiological mechanisms that control growth plate fusion.

## Introduction

Endochondral ossification is a tightly regulated process responsible for the formation and postnatal linear growth of the long bones, including the tibia and femur. Endochondral ossification is carefully orchestrated to involve the replacement of a cartilage scaffold by mineralized bone, and integral to this is the epiphyseal growth plate, a developmental region located in the metaphysis of the long bones (Staines et al., 2012).

The growth plate consists of chondrocytes arranged in columns. These chondrocytes are surrounded by their extracellular matrix, consisting of specific collagens, namely Collagen types II and X, as well as proteoglycans such as aggrecan and other non-collagenous matrix proteins including the SIBLING family of proteins (Ballock and O’Keefe, 2003; Mackie et al., 2011). Chondrocytes undergo distinct maturation stages of proliferation, differentiation, and hypertrophy, while maintaining their spatially fixed location (Hunziker et al., 1987). It is the terminally differentiated hypertrophic chondrocyte, which mineralizes its surrounding extracellular matrix (Castagnola et al., 1988). This process, thought to involve membrane-limited matrix vesicles, is biphasic and tightly regulated by a number of enzymes and factors including alkaline phosphatase (Alpl), PHOSPHO1, the ankylosis protein (Ank), ecto-nucleotide pyrophosphatase/phosphodiesterase-1 (Enpp1) (Terkeltaub et al., 1994; Anderson, 1995; Roberts et al., 2007). Mineralization of the cartilage extracellular matrix facilitates vascular invasion allowing the infiltration of bone resorbing osteoclasts and bone forming osteoblasts (Zelzer et al., 2002). This is a key step in endochondral ossification and enables the replacement of the cartilaginous scaffold by bone.

Throughout the majority of embryonic and postnatal longitudinal bone growth, the processes of mineralized cartilage production and replacement by bone are coupled. However, as growth slows, the human growth plate undergoes progressive narrowing as bony bridges form and span its width. This ultimately leads to complete growth plate closure and the cessation of human growth. These bone bridges are also known to form upon growth plate injury, thought to be through an intramembranous ossification mechanism (Xian et al., 2004). Despite this, the molecular mechanisms underpinning their formation are unknown. It is well established though that growth plate closure in humans coincides with sexual maturation with estrogen playing a pivotal role, although the precise mechanisms are complex and are also yet to be fully established. Indeed, in two genetic mutations resulting in estrogen deficiency (in the estrogen-receptor gene, and in the CYP19 gene), the growth plate fails to fuse and growth persists, albeit rather slowly, into adulthood (Smith et al., 1994; Morishima et al., 1995; Grumbach and Auchus, 1999; Grumbach, 2000).

Whether growth plate fusion occurs prior to or after the cessation of growth is of significant controversy in the field and has been somewhat overlooked (Parfitt, 2002). Evidence from studies in both humans and rats revealed the cessation of growth long before any histological evidence of growth plate fusion, suggesting that epiphyseal fusion is a marker of growth cessation and not its cause (Becks et al., 1948; Moss and Noback, 1958; Weise et al., 2001). This goes against the current state of knowledge and it is essential that the exact temporal and spatial relationship between growth cessation and growth plate fusion is delineated before the complexities of epiphyseal growth plate function can be fully understood.

Research into growth plate fusion mechanisms may have been hindered somewhat by the limitation in current methods available for its visualization, which are invasive and largely reliant upon histological procedures. While nano-scale imaging is now possible *via* many approaches, the high flux and superior detectors in synchrotron (SR) micro-computed tomography (CT) enable excellent spatial resolution and high speed imaging, ideal for the rapid collection of multiple image volumes. Furthermore, computational mechanobiology is recognized as powerful tool for predicting tissue growth and adaptation (Prendergast et al., 1997). The relationship between macroscopic mechanical loads and the local stresses and strains that influence tissue formation can now be calculated using computational models (Carter and Wong, 1988; Lacroix and Prendergast, 2002a,b; Lacroix et al., 2002; Shefelbine and Carter, 2004; Stokes et al., 2006; Garzón-Alvarado et al., 2009; Gerhard et al., 2009; Lin et al., 2009; Reina-Romo et al., 2010; Schulte et al., 2013; Betts and Müller, 2014; Giorgi et al., 2015). To the best of our knowledge, due to the lack of information on the undulating internal structure, the 3D morphology of the growth plate has not yet been included in models of endochondral ossification or its complex fusion mechanisms (Gao et al., 2014). It seems likely that the local mechanical stresses, which may contribute to such fusion will be better predicted by the addition of 3D morphology of the growth plate. Therefore, we have developed a readily accessible method that could be useful in discriminating between normal and abnormal growth plate dynamics.

Here, we describe a non-invasive method for the 3-dimensational (3D) quantification of growth plate bridging using gold standard SR microCT, which we have then also applied to images acquired using a standard, more widely accessible benchtop microCT scanner. We have then used these images to conduct finite element (FE) modeling to assess currently unexplored associations between growth plate bridging and local strain distributions. Exploitation of these new methods is likely to advance understanding of the physiological mechanisms, which lead to growth plate closure and the effects of this on local tissue mechanics.

## Materials and Methods

### Animal Model

Wild-type mice (C57/BL6 and CBA) were housed up to four per cage in polypropylene cages with wood chip and paper bedding and provided standard mouse chow and water *ad libitum* throughout the study. Weaners up to 8 weeks of age were fed a standard rodent breeding diet and thereafter a standard rodent maintenance diet (Special Diet Services, South Witham, UK). All procedures complied with the UK Animals (Scientific Procedures) Act 1986 and were reviewed and approved by the ethics committee of the Royal Veterinary College (London, UK).

### Imaging

#### Tissue Imaging by Benchtop MicroCT

The tibia from a 20-month-old female C57/BL6 mouse (Charles River, UK) was fixed in 70% EtOH and stored until scanning using the Skyscan 1172 (Skyscan, Kontich, Belgium), with X-ray tube operated at 50 kV and 200 μA, 1,600 ms exposure time with a 0.5 mm aluminum filter and an acquisition pixel size of 5 μm. The slices were then reconstructed using NRecon 1.6.9.4 with a pixel matrix of 2,000 × 2,000 (Skyscan, Kontich, Belgium) (Javaheri et al., 2015).

#### Tissue Imaging by SR MicroCT

Tibiae from an 8- and 40-week old CBA mouse were dissected and frozen at −20°C until scanning. The SR microCT was performed at Diamond Light Source on the Diamond-Manchester Branchline I13-2 using 19 keV (based on an energy sensitivity study for optimum image contrast) monochromatic X-ray (Rau et al., 2011). 1,800 projections on 180° were collected using a 4× magnification lens by a PCO 4000 CCD imaging camera with 4,008 × 2,672 pixels, giving an effective pixel size of 1.1 μm. The projections were normalized and reconstruction was performed with the tomography reconstruction module of the software Data Analysis WorkbeNch (Dawn) 1.7 (Basham et al., 2015), using Diamond’s computing cluster to produce 3D volumes of the X-ray attenuation (Atwood et al., 2010; Titarenko et al., 2010). The images were filtered using a 3D median filter with a small kernel size of 2 to remove the “shot noise” common with tomography with minimal blurring effect on image texture.

#### Development of a 3D Quantification Method for Growth Plate Bridging

**Figure 1:**
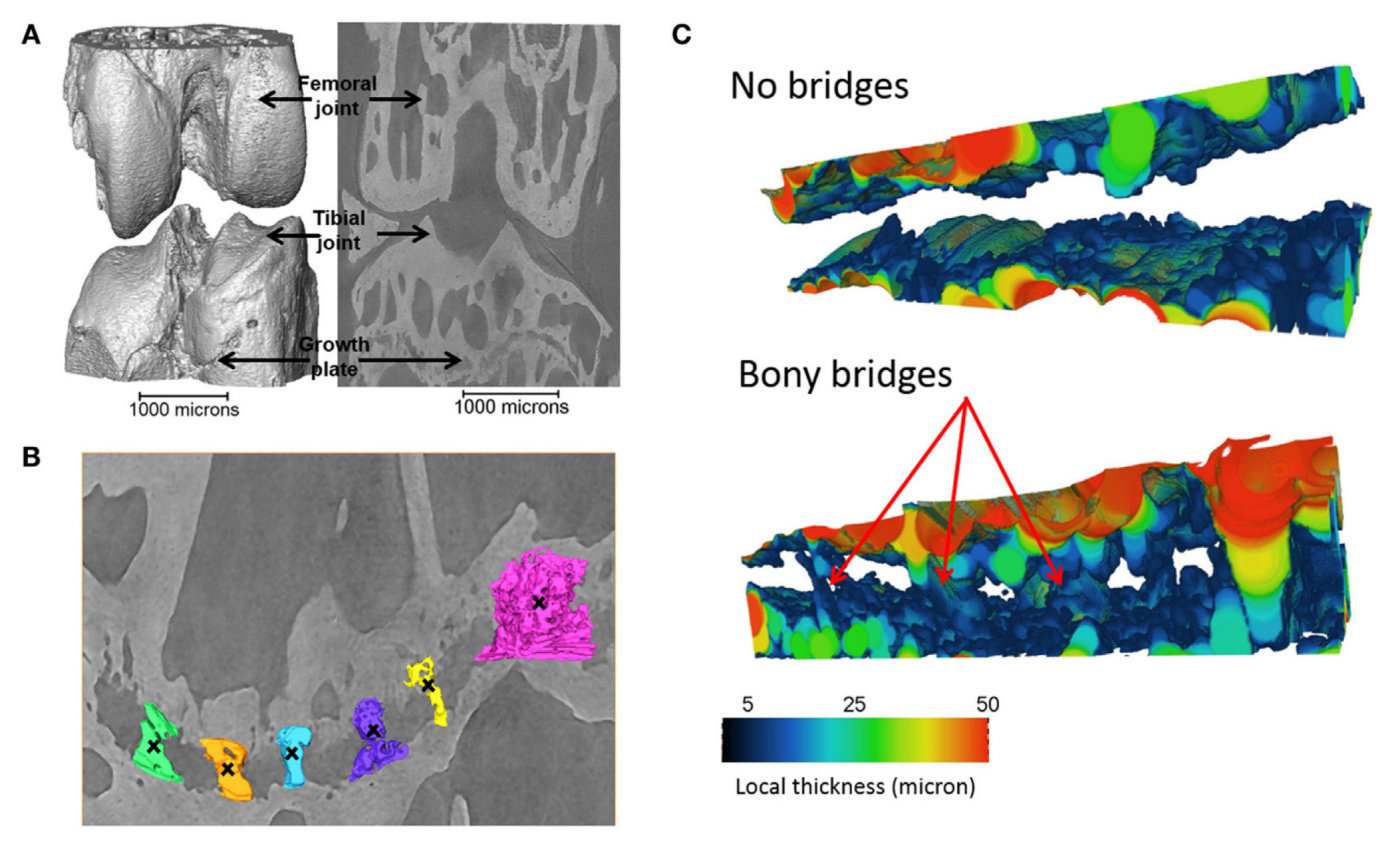
Identification of the bridges crossing the growth plate **(A)** 3D representation of the whole joint, **(B)** bridges crossing the growth plate, in an ROI (black crosses indicate bony bridges identified by an observer). **(C)** 3D representation of the growth plate with no bridges and multiple bridges observed. Adapted from Staines et al. (2016).

**Figure 2:**
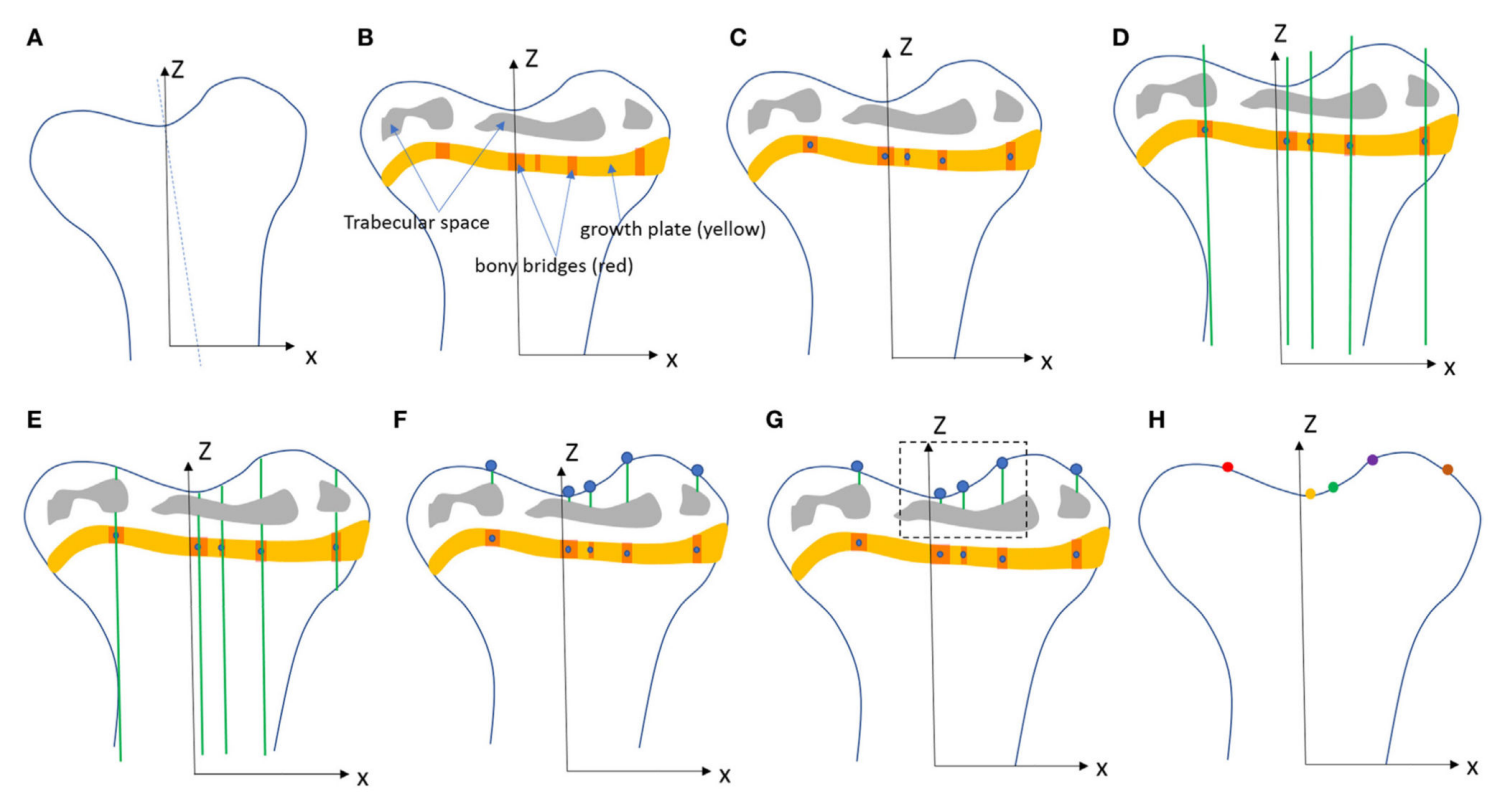
Procedure developed to project the bridges and their areal number density onto the tibial joint surface: **(A)** the volume images were aligned along the metaphyseal tibial shaft (*z*-axis), **(B)** the central point of all bony bridges crossing the growth plate were examined from slices cut in multiple orientations, and identified by an observer who highlighted them individually with the cursor, **(C)** the center of all bridges (blue circles) were extracted using a skeletonization, **(D)** generation of straight lines along the (*z*) direction passing through the bridges, **(E)** intersection between the straight lines and the tibia (segmented using a region growing algorithm), **(F)** detection of endpoints of the lines that intercept the joint surface, **(G)** the areal number density, *N*_A_, is calculated as the number of bridges per 256 μm × 256 μm window, **(H)** the distribution of the areal number density of bridges is superimposed on the tibial joint surface (each bridge has a color that represents the areal number density at the bridge location).

Tibiae scans from both benchtop microCT or SR microCT were used to identify growth plate bridges observed as indicated in Figure 1. Scans were segmented using Avizo^®^ software (V8.0, VSG, Burlington, VT, USA), using a region-growing algorithm within the software. This algorithm starts from a seed point and selects all connected voxels with a gray value in a given tolerance interval. The volume images were aligned manually along the metaphyseal tibial shaft (defined as the *z*-axis; Figure 2A) and the central points of each individual bony bridge crossing the entire growth plate width were examined from slices cut in multiple orientations, and individually identified by an observer (Figure 2B). To reduce uncertainties due to partial volume effects, all the bridges smaller than about 125 voxels in volume were not considered. Once all the bridges were selected, each was quantified and rendered a separate color to confirm correct manual identification.

**Figure 3:**
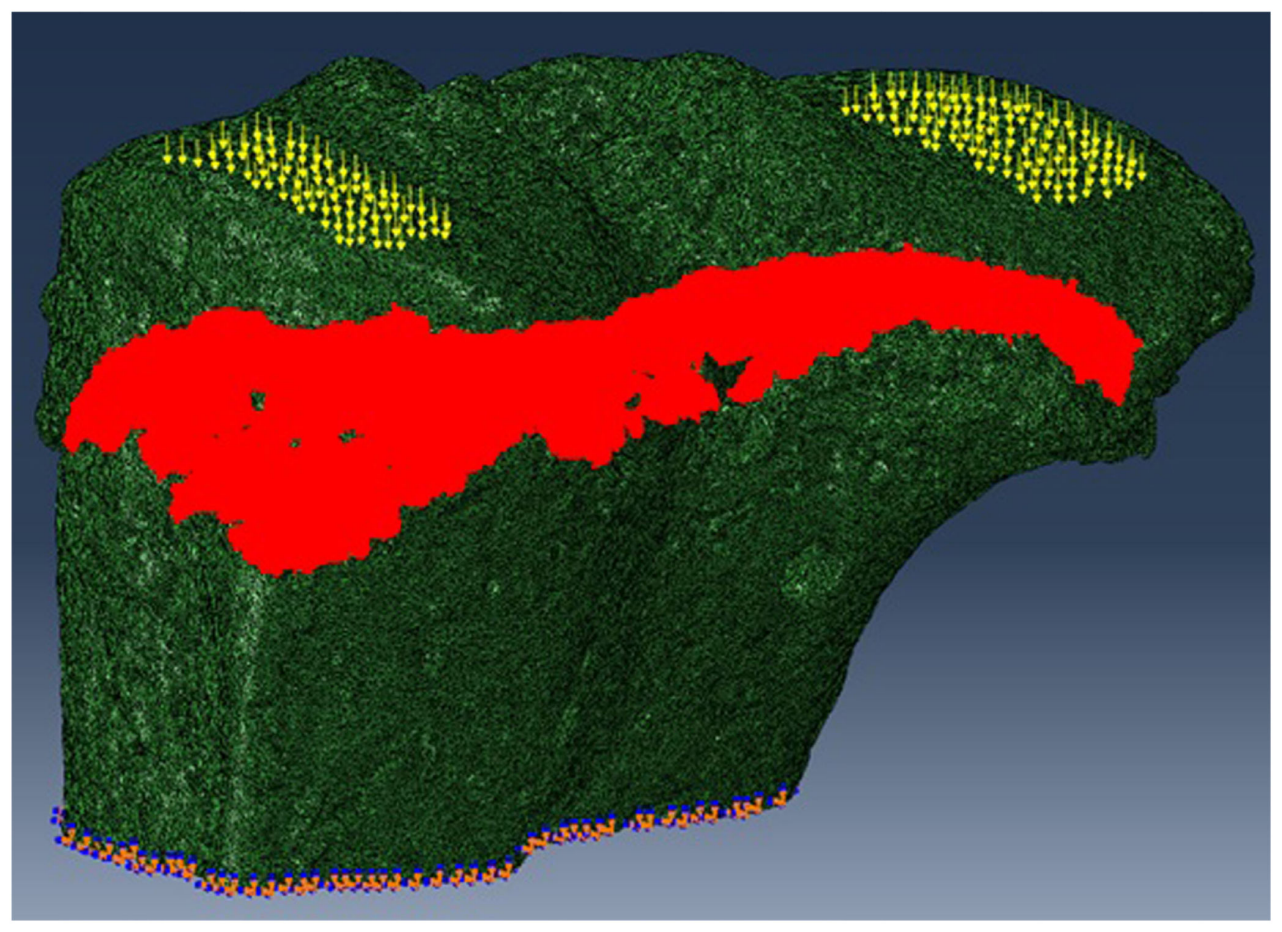
For finite element (FE) modeling, FE computations were carried out to simulate static compressive tests (sustained loading). The nodes at the bottom of the tibia have their vertical displacements fixed and certain rotations are restrained to block rigid body motion. At the top, equal loads of three times body weight (~1 N) were applied in the medial and lateral aspects of the tibia.

To quantify the bone bridge local number density, a method for projecting them onto the joint surface was developed. The center of all bridges (blue circles) were extracted using a skeletonization (Figure 2C) and each bridge was orthogonally projected onto the tibial joint surface using an in-house line intercept method implemented in Matlab (Mathworks, USA). The method consists of (1) generating straight lines along the (*z*) direction passing through the bridges (Figure 2D), (2) determining the intersection between the straight lines and the tibial plateau (segmented using a region growing algorithm, Figure 2E), and (3) detecting the endpoints of the lines that intercept the joint surface (Figure 2F). From this, the areal number density, *N*, is defined as the number of bridges per 256 μm × 256 μm window (Figure 2G). The distribution of the areal number density of bridges is then superimposed on the tibial joint surface (each bridge has a color that represents the areal number density at the bridge location, Figure 2H) (Staines et al., 2016).

#### FE Modeling

The SR microCT images from the 8- and 40-week old mice were imported into the Avizo software (Avizo Fire, 9.2.0, VSG) to generate unstructured linear tetrahedral meshes using the methodology described by Madi et al. (2007). FE computations were carried out to simulate static compressive tests (sustained loading). The nodes at the bottom of the tibia have their vertical displacements fixed and certain rotations have been restrained to block rigid body motion. To restrain the rotations, one node is blocked in the three directions, one node is blocked along *X* and one node is blocked along *Y*. At the top, loads equal to three times body weight (~1 N) were applied as nodal forces (Cook et al., 1983) in the medial and lateral aspects of the tibia (Figure 3). Bone and growth plate cartilage tissues were treated as isotropic linear elastic materials (see Table 1) (Piszczatowski, 2012; Poulet et al., 2013). Following a mesh sensitivity study, the number of elements was fixed to about 2,500,000. The mesh density was approximately 250 voxels/element (the images were binned, i.e., 2.2 μm voxel size), and was based on average and local (line profiles) axial stresses plotted against number of elements (error criterion: 5% of asymptotic value).

## Results

Herein, we used our method for the visualization and quantification of growth plate bridging, and our FE model to examine the effects of bridging on local tissue mechanics.

### Application of Novel Method to Benchtop MicrocT Scans

**Figure 4:**
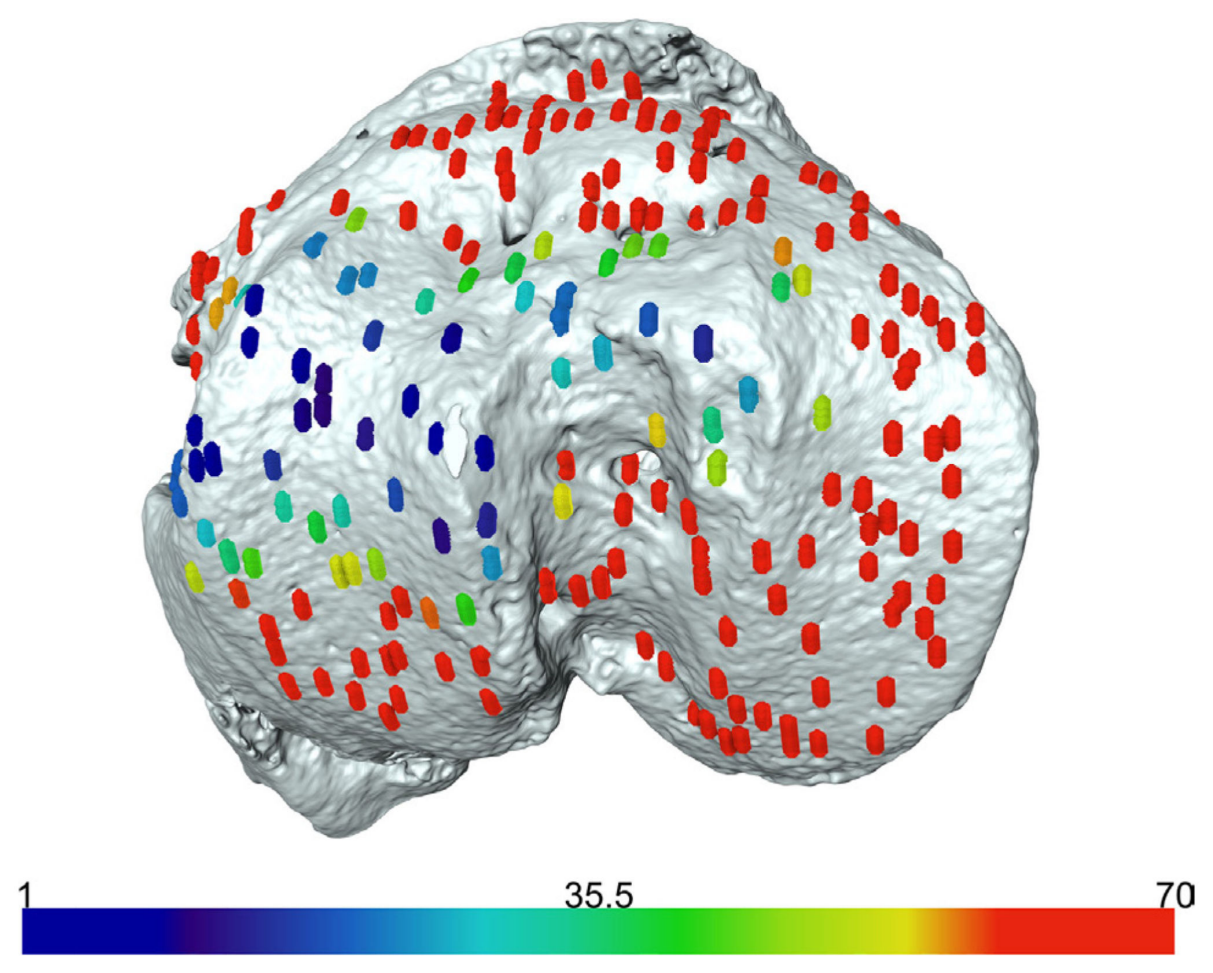
Laboratory-based computed tomography image of the tibial joint surface from a 20-month-old C57BL6 female mouse. The distribution of the areal number density of bridges is superimposed on the tibial joint surface and each bridge has a color that represents the areal number density at the bridge location.

We have previously shown that the above developed method is a valuable model for examining growth plate bridging during healthy and pathological aging (Staines et al., 2016). However, the use of SR microCT is not readily available for most laboratories due to high costs and availability of beamtime. Therefore we sought to examine whether our method could be applied to benchtop microCT at a pixel size of 5 μm. As detailed in our methods, the tibia from a C57/BL6 20-month-old female mouse was scanned using the SkyScan 1172 scanner. Images were reconstructed and our method for growth plate bridging 3D quantification method was applied (Figures 1 and 2). We were able to clearly identify growth plate bridges at 5 μm and once all the bridges were selected, we were able to superimpose the distribution of the areal number density onto the tibial joint surface (Figure 4). Spatial evaluation of growth plate bridges indicates that thicker bridges are not randomly distributed but rather that these are preferentially enriched in peripheral growth plate locations, consistent with our previous results (Staines et al., 2016) (Figure 4).

### Application of 3D Quantification of Growth Plate Bridging in FE Models of the Loaded Tibia

**Figure 5:**
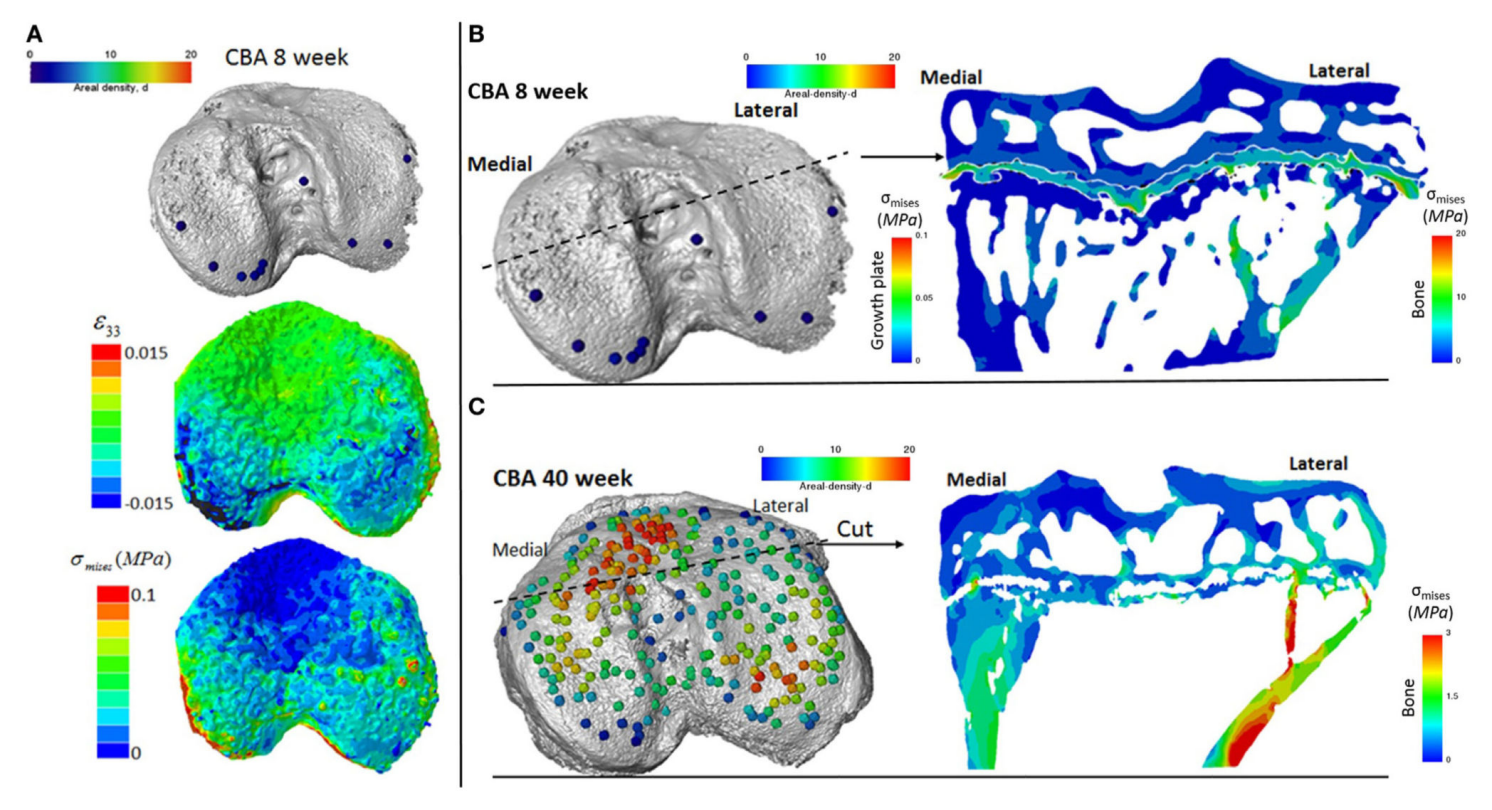
Finite element models of the loaded tibia in CBA wild-type mice **(A)** visualization of von Mises stresses on the tibial joint plateau. Visualization of the stresses associated with growth plate bridging in a coronal section of the tibia for a **(B)** young (8 week) and **(C)** mature (40 week) mouse joint.

We reveal that in young (8 weeks old) CBA wild-type mice, growth plate bridging is associated with locations that contain high local von Mises stresses (Figures 5A,B). Moreover, we reveal that with aging an increased number and density of growth plate bridges is observed (Figure 5B), indicative of growth plate closure (Staines et al., 2016). Our FE modeling indicates that this increase in growth plate bridging with aging is translated into greater stresses in the growth plate (Figure 5C). This, therefore, offers insights into the biomechanical functionality of growth plate fusion.

## Discussion

The results presented in this study report a method for the 3D quantification of growth plate bridging in murine bones. Although initially developed for SR microCT, we demonstrate that this method can also be applied to benchtop microCT scans collected at high resolutions. Furthermore, we reveal that this model can then be combined with FE modeling to understand local tissue mechanics. The relationship between mechanical loads and the local stresses and strains that influence tissue formation can be calculated using computational models for macro-, meso-, and nano-scales (Webster et al., 2008; Schulte et al., 2013). This method described herein will contribute to future advances in the development of hierarchical models for understanding bone development and adaptation.

We show that, in wild-type mice, increased growth plate bridging translates into increased stresses in the bone directly beneath the growth plate. At 8 weeks (Figure 5B), few bridges are detected and overall the growth plate is squeezed in a “sandwich” configuration. This suggests that compressive hydrostatic stresses are engendered across major volumes and that higher shear stresses are generated only at the peripheral edges of the growth plate. Yet, the results of numerous mechanobiological models support that growth and ossification is accelerated by tensile strain (or shear stresses) and that cartilage tends to be maintained by hydrostatic compressive stress (Carter and Wong, 2003; Stokes et al., 2007; Villemure and Stokes, 2009). This would be consistent with our observations and may offer an explanation for why the bridges start growing first at the edges. At 40 weeks (Figure 5C), the mechanical environment of the growth plate is more complex; the thicker bridges act as stress concentrators. This appears to increase the likelihood that growth plate bridges in older animals will fracture under loading and/or achieve the redistribution of stresses to within their particular vicinities of the growth plate cartilage. Reciprocally, during endochondral ossification, growth-related strain in the mice may generate stresses that are contained by the bony bridges, accelerating growth arrest at these vicinities. More time points (between 8 and 40 weeks) and modeling are needed to support this hypothesis, but this work has provided novel insights into the use of CT, advanced image processing, and FE modeling in understanding biomechanics in health and disease. Our methodology offers a unique mode for visualizing these events and the potential to address questions that have hitherto remained elusive in this field.

There are a number of unanswered questions in our pursuit of understanding growth plate closure such as; in which direction does growth plate bridging occur? Does bridging follow a conserved pattern across all growth plates? What is the trajectory of individual bridge expansion during growth cessation? And does genetics or mechanics define the initiation and progression of growth plate bridging? Such questions may now be addressable with the development of our novel non-invasive method for 3D quantification of bony bridging linked to FE models, and this will likely advance understanding of mechanisms involved in growth plate closure, which have hitherto been hindered somewhat by reliance upon invasive, largely histological methods.

The growth plate is responsible for the development and growth of long bones, up until puberty at which point it begins to close. During this closure, bone bridges form and span the width of the growth plate, eventually leading to the replacement of the entire growth plate cartilage anlagen by mineralized bone. Histological studies have identified this to be an early event involving several pre-osteoblastic molecules such as osteoprotegerin, interleukin-6, bone morphogenetic protein, and Collagen type X (Pichler et al., 2013). Similarly, trauma to the growth plate, e.g., in fractures, can also provoke abnormal growth plate bridging and the impairment of longitudinal growth.

Biomechanically, the growth plate is subject to a number of different loads placed upon it and previous studies have identified regional variations in the mechanical properties of the growth plate and surrounding tissues by confocal and atomic force microscopies (Bachrach et al., 1995; Radhakrishnan et al., 2004; Villemure et al., 2007). It is known that different compressive mechanical strains are found throughout the different zones of the growth plate, with the proliferative zone of chondrocytes exhibiting lower compressive strains than that of the resting and hypertrophic zones (Villemure et al., 2007). Similarly, compressive differences are also observed spatially throughout the growth plate with interior samples from bovine femoral growth plates being 40% more rigid than samples taken from the periphery of the growth plate (Cohen et al., 1994). Here, we reveal an association between growth plate bridging and increased stress dissipation distal to the growth plate, by applying FE modeling to our method. Future investigations will improve FE models to include more realistic boundary conditions, material properties, and cartilage mechanobiological principles. Our final aim is to examine the combined effects of hydrostatic pressure and shear stress (in the form of an osteogenix index) on the development of the skeleton and articular cartilage.

We have previously used SR microCT to study growth plate bridging in the context of a murine model of spontaneous osteoarthritis (Staines et al., 2016). Herein, we have also employed benchtop microCT to image the tibia of wild-type mice and confirm suitability of using benchtop microCT-based imaging for bridging analysis. Despite imaging at a lower resolution (5 instead of 1.1 μm voxel size), we were able to replicate our method and confirm that in both imaging modalities, similar data can be obtained. Further, the data obtained from the bench-top microCT are analogous to the gold-standard SR imaging. Of course, there are disadvantages related to the use of dose radiation. This may have an impact on the mechanical properties of bone and cartilage and limit the validity of 4D *in situ* mechanical experiments as showed by Barth et al. (2010), but to our knowledge, exert little effect on the 3D morphology. The missing link in current mechanobiological models is the 3D real morphology of the growth plate which is very complex and cannot be captured correctly by 2D approaches. Herein, we have aimed to address this by describing and then utilizing our 3D non-destructive imaging methodology for the quantification of growth plate bridging. Employment of these 3D analyses of growth plate bone bridging is likely to advance our understanding of the physiological mechanisms, which lead to growth plate closure and subsequent FE modeling allows investigations into the associated biomechanical functionality. This will ultimately enable investigators to study the role of local tissue mechanics on endochondral ossification patterns, skeletal morphology, and articular cartilage function.

## Figures and Tables

**Table 1:** Material properties of bone and cartilage.

Material properties	Bone	Growth plate
*E* (MPa)	17,000	6
Poisson coefficient	0.3	0.49
